# Elevated concentrations of serum matrix metalloproteinase-2 and -9 and their associations with circulating markers of cardiovascular diseases in chronic arsenic-exposed individuals

**DOI:** 10.1186/s12940-015-0079-7

**Published:** 2015-12-04

**Authors:** Md Shofikul Islam, Nayan Chandra Mohanto, Md Rezaul Karim, Sharmin Aktar, Md Mominul Hoque, Atiqur Rahman, Momotaj Jahan, Rabeya Khatun, Abdul Aziz, Kazi Abdus Salam, Zahangir Alam Saud, Mostaque Hossain, Aminur Rahman, Abul Mandal, Azizul Haque, Hideki Miyataka, Seiichiro Himeno, Khaled Hossain

**Affiliations:** Department of Biochemistry and Molecular Biology, University of Rajshahi, Rajshahi-6205, Bangladesh; Department of Applied Nutrition and Food Technology, Islamic University, Kushtia-7003, Bangladesh; Infectious Disease and Immunogenetics Section, Department of Transfusion Medicine, Clinical Center, National Institutes of Health, Bethesda, MD 20892 USA; Kaliganj Upazila Health Complex, Gazipur, Dhaka Bangladesh; Systems Biology Research Centre, School of Bioscience, University of Skövde, Skövde, Sweden; Department of Microbiology and Immunology, Medical University of South Carolina, Charleston, SC USA; Laboratory of Molecular Nutrition and Toxicology, Faculty of Pharmaceutical Sciences, Tokushima Bunri University, Tokushima 770-8514, Japan

**Keywords:** Arsenic, MMP-2, MMP-9, Cardiovascular diseases, Cancer, Bangladesh

## Abstract

**Background:**

Cardiovascular diseases (CVDs) and cancers are the major causes of chronic arsenic exposure-related morbidity and mortality. Matrix metalloproteinase-2 (MMP-2) and −9 (MMP-9) are deeply involved in the pathogenesis of CVDs and cancers. This study has been designed to evaluate the interactions of arsenic exposure with serum MMP-2 and MMP-9 concentrations especially in relation to the circulating biomarkers of CVDs.

**Methods:**

A total of 373 human subjects, 265 from arsenic-endemic and 108 from non-endemic areas in Bangladesh were recruited for this study. Arsenic concentrations in the specimens were measured by inductively coupled plasma mass spectroscopy (ICP-MS) and serum MMPs were quantified by immunoassay kits.

**Results:**

Serum MMP-2 and MMP-9 concentrations in arsenic-endemic population were significantly (*p* < 0.001) higher than those in non-endemic population. Both MMPs showed significant positive interactions with drinking water (*r*_*s*_ = 0.208, *p* < 0.001 for MMP-2; *r*_*s*_ = 0.163, *p* < 0.01 for MMP-9), hair (*r*_*s*_ = 0.163, *p* < 0.01 for MMP-2; *r*_*s*_ = 0.173, *p* < 0.01 for MMP-9) and nail (*r*_*s*_ = 0.160, *p* < 0.01 for MMP-2; *r*_*s*_ = 0.182, *p* < 0.001 for MMP-9) arsenic of the study subjects. MMP-2 concentrations were 1.02, 1.03 and 1.05 times, and MMP-9 concentrations were 1.03, 1.06 and 1.07 times greater for 1 unit increase in log-transformed water, hair and nail arsenic concentrations, respectively, after adjusting for covariates (age, sex, BMI, smoking habit and hypertension). Furthermore, both MMPs were increased dose-dependently when the study subjects were split into three (≤10, 10.1-50 and > 50 μg/L) groups based on the regulatory upper limit of water arsenic concentration set by WHO and Bangladesh Government. MMPs were also found to be significantly (*p* < 0.05) associated with each other. Finally, the concentrations of both MMPs were correlated with several circulating markers related to CVDs.

**Conclusions:**

This study showed the significant positive associations and dose–response relationships of arsenic exposure with serum MMP-2 and MMP-9 concentrations. This study also showed the interactions of MMP-2 and MMP-9 concentrations with the circulating markers of CVDs suggesting the MMP-2 and MMP-9 -mediated mechanism of arsenic-induced CVDs.

## Background

Arsenic is a potent environmental pollutant and a well established human carcinogen. Chronic exposure to arsenic is associated with a variety of diseases such as cancers, dermatitis, CVDs, neurological disorders, diabetes mellitus, renal failure and liver dysfunction [1–9]. Exposure to arsenic through drinking water is a major threat to the public health in many countries especially in Bangladesh, India, Taiwan, China, Vietnam, Cambodia and Mongolia. Arsenic poisoning has turned into tragedy causing thousands of deaths in Bangladesh. About 80–100 million people are at risk of arsenic toxicity in the country because of the consumption of higher concentrations of arsenic through drinking water as compared to the permissive limit set by World Health Organization (WHO). Recent reports suggest that arsenic has entered the food chain including rice and vegetables [10, 11]. Presence of excessive amount of arsenic in foods and vegetables indicate that exposure to arsenic is unavoidable.

Matrix metalloproteinases (MMPs) are a family of zinc-dependent proteolytic enzymes that degrade various components of extracellular matrix (ECM). ECM and their components are organized by a complex structure of collagens, elastins, gelatins, laminins, fibronectins, and proteoglycans. Rigorous regulation of MMP production and activation is a crucial part of the homeostasis of ECM. Impaired collagen and ECM homeostasis may be among the underlying molecular mechanisms that lead to the development of CVDs and cancers. Among the MMPs, MMP-2 (gelatinase A) and MMP-9 (gelatinase B) have been widely studied that degrade both gelatins and collagens of ECM. MMP-2 and MMP-9 activities are largely regulated by tissue inhibitor of metalloproteinase-1 (TIMP-1) [12, 13]. These MMPs have been implicated in cardiovascular and cancer pathology through the degradation of ECM. In cardiovascular pathology, elevated MMP activities have been reported to be linked to atherosclerotic plaque formation and plaque instability [14, 15]. In cancer pathology, MMP-2 and MMP-9 are mainly implicated in the formation of new blood vessels through angiogenesis. MMP-2 and MMP-9 facilitate the migration of tumor cells to blood vessels by the degradation of basement membrane ECM proteins. CVDs and cancers are the major causes of chronic arsenic exposure-related morbidity and mortality, however, underlying mechanisms of these arsenic-induced diseases remains to be clarified. Although MMPs are deeply implicated in several chronic diseases, previously only a limited number of human studies have evaluated the effect of chronic arsenic exposure on MMPs [16–19]. Furthermore, the interaction of MMPs with arsenic-induced chronic diseases especially CVDs have not yet been documented clearly. Therefore, this study has been designed to evaluate the interactions of arsenic exposure with serum MMP-2 and MMP-9 concentrations in humans especially in relation to the circulating biomarkers of CVDs.

## Methods

### Study areas and study subjects

Ethical permission was taken from the Institute of Biological Sciences, University of Rajshahi, Bangladesh (21/320-IAMEBBC/IBSc). The human subjects who participated in this study gave their written consent. Confidentialities and rights of the study subjects were strictly maintained. Arsenic-endemic and non-endemic study areas for this study were selected as we described previously [3, 9, 20–24]. Arsenic-endemic areas were selected from the north-west region of Bangladesh that included Marua in Jessore, Dutpatila, Jajri, Vultie and Kestopur in Chuadanga, and Bheramara in Kushtia district of Bangladesh. Chowkoli, a village in Naogaon district with no history of arsenic contamination was selected as a non-endemic area. Local residents (15–60 years of ages) who had lived for at least five years in arsenic-endemic and non-endemic areas were recruited for this study. The study subjects were selected from the convened group, irrespective of the presence or absence of arsenic-induced skin symptoms (melanosis, leukomelanosis and keratosis). Subsequently, individuals who did exhibit skin symptoms were first identified by a physician and then the diagnosis was confirmed by a dermatologist. The physician involved in this study carefully examined various parts of the body to confirm the presence of skin lesions.

During the sample collection process, we were blinded to arsenic concentrations in the drinking water, and to those in the hair and nails of the study participants. Attempts were made to match, as much as possible age, sex and socioeconomic parameters (occupation, monthly income and education) in the two population groups selected from arsenic-endemic and non-endemic areas. The ratio of male to female study subjects was approximately 1:1.

Pregnant and lactating mothers, and the individuals who had previous and recent history of surgical operation, drug addiction, hepatitis B positive, hepatotoxic and anti-hypertensive drugs, malaria, kalazar, chronic alcoholism, previous and present history of hepatic, renal or severe cardiac diseases have been excluded from this study. Of the 274 individuals who were approached, 9 were excluded according to the exclusion criteria [i.e., study candidates (*n* = 4) who had reside in arsenic-endemic areas for less than 5 years, pregnant and lactating mothers (*n* = 3), and had hepatic diseases (*n* = 2)]; thus a total of 265 individuals were finally recruited in arsenic-endemic areas. In non-endemic area, four [i.e., study candidates (*n* = 1) who had resided in the non-endemic area for less than 5 years, pregnant and lactating mothers (*n* = 2), study subjects who underwent recent surgical operation (*n* = 1)] from a total of 112 individuals were excluded. The number of final participants was 108 in non-endemic area.

Household visits were carried out to interview residents. Personal interview of the study subjects was carried out by the trained members of our research team using a standard questionnaire. Information obtained from the interview included the sources of water for drinking and daily house hold uses, water consumption history, socioeconomic status, occupation, food habit, general food items consumed daily, cigarette smoking, alcohol intake, personal medical history, history of diseases, physiological complications, major diseases, previous physician’s reports, and BMI. We collected all blood and other specimens (including water samples) on the same day at each site.

### Blood pressure measurement

The standard protocol for measuring blood pressure recommended by WHO, was used in this study. After study subjects had rested for 20 min or longer, both systolic and diastolic blood pressures (SBP and DBP) were measured three times with a mercury sphygmomanometer with subjects sitting. SBP and DBP were defined at the first and fifth phase Korotkoff sounds, respectively. The average of three measurements was used for the analysis. Hypertension was defined as a SBP of ≥ 140 mm Hg and a DBP of ≥ 90 mm Hg on three repeated measurements.

### Collection of serum and examination for Hepatitis B

All study subjects were requested to fast overnight (10–12 h), and fasting blood samples (5–7 ml) were collected from each individual by venipuncture into blood collection tubes. The blood samples were left at room temperature for 30 min for clotting, and were subsequently centrifuged at 1200 × g for 20 min. The serum supernatant was then taken and stored at -80 °C. All serum samples were checked for hepatitis B using a 3^rd^-generation HBsAg ELISA test kit (Medivent Diagnostic & Co. Ltd., Ireland) according to the manufacturer’s protocol.

### Water collection and arsenic analysis

Tube well water identified by study subjects as primary sources of their drinking water were collected for this study as described by Ali et al. [23]. Water samples from tube wells were collected in acid-washed containers after the well was pumped for 5 min as described previously [25]. Total arsenic concentrations in water samples were determined by inductively coupled plasma mass spectroscopy (ICP-MS), (HP-4500, Agilent Technologies, Kanagawa, Japan) after the addition of a solution of yttrium (10 ppb in 1.0 % nitric acid) to all water samples as an internal standard for ICP-MS analysis. All samples were determined in triplicate and the average values were used for data analysis. Accuracy of water arsenic measurement was verified using a certified reference material (CRM). “River water” (NMIJ CRM 7202-a No.347 National Institute of Advanced Industrial Science and Technology, Japan) was used as a CRM. The average value (mean ± SD) of arsenic in the “river water” determined in triplicate by ICP-MS analysis was 1.06 ± 0.04 μg/L (reference value, 1.18 μg/L).

### Collection of hair and nail samples, and analysis of arsenic

Arsenic concentrations in finger nails and hair have been reported to provide the integrated measures for arsenic exposure [26, 27]. Hair and nails of the study subjects were collected and washed by the method as described previously [23]. The washed samples were allowed to dry at 60 °C overnight and digested with concentrated nitric acid using a hot plate at 70 °C for 15 min and 115 °C for 15 min. After cooling, the samples were diluted with 1.0 % nitric acid containing yttrium (10 ppb). The concentrations of arsenic and yttrium in these samples were determined by ICP-MS. All samples were determined in triplicate and the average values were used. Accuracy of arsenic measurement was verified using “human hair” (GBW09101, Shanghai Institute of Nuclear Research Academia Sinica, China) as a CRM. The average value (mean ± SD) of arsenic in “human hair” determined in triplicate by ICP-MS analysis was 0.61 ± 0.12 μg/g (reference value, 0.59 μg/g).

### Measurements of serum MMP-2 and MMP-9

Serum concentrations of MMP-2 and MMP-9 were measured using commercially available enzyme-linked immunoassay kits (MMP-2 from R&D Systems, Inc. Minneapolis, USA, and MMP-9 from Invitrogen Corporation, Camarillo, CA) according to the manufacture’s protocols. A micro-plate reader (Mikura Ltd. UK) was used for the measurement of color development. All standards and samples were analyzed in duplicate. The intra and inter assay coefficients of variations (CVs) were maximum 10 %.

### Statistical analysis

Descriptive statistics were calculated to compare the study subjects of non-endemic and arsenic-endemic areas by independent sample t-test for continuous variables, and chi-square test for categorical variables. Variables (MMP-2, MMP-9, water, hair and nail arsenic) were log-transformed to improve the approximation to the normal distribution. Normality of the variables distribution was verified by a Q-Q plot. Comparisons of MMP-2 and MMP-9 concentrations between the two population groups in arsenic-endemic and non-endemic areas were analyzed by independent sample t-test. Spearman correlation coefficient tests were used to evaluate the correlations of MMP-2 and MMP-9 concentrations with arsenic exposure metrics (water, hair and nail arsenic). The nature of any association between arsenic exposure metrics and MMPs was evaluated by scatter plot analysis. Study subjects in the arsenic-endemic areas were split into two (medium and high) groups based on the two concentrations (water, hair and nail) of each exposure metric with equal proportion through frequency test, and study subjects in the non-endemic area were used as a reference group (low exposure group). MMP-2 and MMP-9 concentrations for the low, medium and high arsenic exposure metrics were analyzed by general linear model univariate regression followed by Bonferroni multiple comparison tests. We estimated the ratios of the geometric means of MMP-2 and MMP-9 which were interpreted as exponentiated regression coefficients. The linear model univariate regression analyses were performed before (Model 1) and after (Model 2) adjusting for relevant covariates (age, sex, BMI, smoking and hypertension). Study subjects were further split into three (≤10 μg/L, 10.1-50 μg/L and > 50 μg/L) groups based on the regulatory upper limit for water arsenic concentrations set by WHO (10 μg/L) and Bangladesh Government (50 μg/L). MMP-2 and MMP-9 concentrations in the three groups were evaluated by one way ANOVA test (Bonferroni). Spearman correlation coefficient test was used to evaluate the correlation between serum MMP-2 and MMP-9 concentrations. Correlations of MMP-2 and MMP-9 with high density lipoprotein cholesterol (HDL-C), intercellular adhesion molecule-1 (ICAM-1) and vascular cell adhesion molecule-1 (VCAM-1) were evaluated by Spearman correlation coefficient test. Statistical analysis for this study was done with Statistical Package for the Social Sciences (SPSS version 17.0, SPSS Inc., Chicago, IL). A value of *p* < 0.05 was considered statistically significant.

## Results

Table 1 shows some general characteristics of the study populations in arsenic-endemic (*n* = 265) and non-endemic areas (*n* = 108). There were 145 male and 120 female study subjects in arsenic-endemic areas, and these were 53 and 55, respectively, in non-endemic area. Since attempts were made to match, as much as possible age, sex and socioeconomic parameters (occupation, education and monthly income) between the two population groups in arsenic-endemic and non-endemic areas, no significant differences were observed in those parameters between the two study populations. The average ages (mean ± SD) of the arsenic-endemic and non-endemic study populations were 38.32 ± 11.79 years and 35.84 ± 11.17 years, respectively. A good number of male and female study subjects were farmers and housewives, respectively, in both arsenic-endemic and non-endemic areas. More than 50 % of the study subjects in arsenic endemic and non-endemic areas had no formal education. The average (mean ± SD) monthly incomes of the study subjects in both arsenic-endemic and non-endemic areas were almost similar. The average (mean ± SD) levels of systolic blood pressure (SBP) and diastolic blood pressure (DBP) of the study population in arsenic-endemic areas were significantly (*p* < 0.001) higher than those in non-endemic area. Accordingly numbers of hypertensive study subjects were also higher in arsenic-endemic areas than those in non-endemic area. A large number (69.43 %) of study subjects in arsenic-endemic areas had typical skin symptoms of arsenicosis. No female was found to be a smoker since generally women in Bangladesh do not smoke cigarette. None of the study subjects admitted to drinking alcohol. Bangladeshi people generally do not drink alcohol because of religious and social restrictions on alcohol drinking. The average levels of BMI (mean ± SD) were almost similar between the two population groups. The range of drinking water arsenic concentrations in the study populations in arsenic-endemic and non-endemic areas were 0.11–546 μg/L and 0.03–13.17 μg/L, respectively. The 25^th^, 50^th^, 75^th^, 90^th^ percentile values of water arsenic in arsenic-endemic individuals were 36.1, 133.8, 263, and 400 μg/L, respectively, where as these were 0.04, 1.18, 2.98 and 5.32 μg/L, respectively, in non-endemic individuals. Arsenic concentrations in the drinking water, hair and nails of the study subjects in arsenic-endemic areas were approximately 136, 12 and 8 times higher, respectively than those in non-endemic area.

**Table 1:** Descriptive characteristic of the study populations in arsenic-endemic and non-endemic areas

Parameters		Non-endemic	Arsenic-endemic	*p*-value
Total subjects (n)		108	265	
Sex (n)	Male	53	145	
	Female	55	120	
Age (years)^a^		35.84 ± 11.17	38.32 ± 11.79	0.062^*^
Occupation [n, (%)]	Male			0.259^†^
	Farmers	37 (69.81)	126 (86.90)	
	Business	0	6 (4.14)	
	Students	13 (24.53)	4 (2.76)	
	Tailors	0	2 (1.38)	
	^+^Others	3 (5.66)	7 (4.83)	
	Female			
	Housewives	53 (96.36)	114 (95.00)	
	Farm workers	2 (3.64)	2 (1.67)	
	Students	0	3 (2.50)	
	^‡^Others	0	1 (0.83)	
Education [n, (%)]	No formal education	62 (57.41)	143 (53.96)	0.397^†^
	Primary	39 (36.11)	93 (35.09)	
	Secondary	5(4.63)	26 (9.81)	
	Higher	1 (1.85)	3 (1.32)	
Income/month (US$)^a^		22.95 ± 5.54	24.33 ± 8.86	0.071^*^
Systolic blood pressure (mmHg)^a^		110.1 ± 14.5	118.26 ± 15.21	<0.001^*^
Diastolic blood pressure (mmHg)^a^		69.81 ± 9.57	76.70 ± 9.60	<0.001^*^
Hypertension [n, (%)]	Yes	2 (1.85)	35 (13.20)	<0.01^†^
	No	106 (98.15)	230 (86.80)	
Skin symptom [n, (%)]	(+) symptom	0	184 (69.43)	<0.001^†^
	(−) symptom	108 (100)	81 (30.57)	
Smoking in male [n, (%)]	Yes	20 (37.74)	59 (40.69)	0.707^†^
	No	33(62.26)	86 (59.31)	
Alcohol intake		-	-	-
BMI (kg/m^2^)^a^		21.13 ± 2.78	20.53 ± 3.15	0.090^*^
Distribution of drinking water As (μg/L)	Range (min-max)	0.03–13.17	0.11–546	
	25^th^ percentile	0.04	36.1	
	50^th^ percentile	1.18	133.8	
	75^th^ percentile	2.98	263	
	90^th^ percentile	5.32	400	
Drinking water As (μg/L)^b^		0.56 ± 7.77	76.10 ± 5.91	<0.001^*^
Hair As (μg/g)^b^		0.23 ± 2.36	2.85 ± 2.97	<0.001^*^
Nail As (μg/g)^b^		0.80 ± 2.64	6.60 ± 2.49	<0.001^*^

Abbreviations: *As* arsenic. BMI was calculated as body weight (Kg) divided by height squared (m^2^). ^a^Mean ± SD. ^b^Geometric Mean (GSD). *Independent sample t-test and ^†^chi-square test were used to test for sample differences in continuous and categorical variables, respectively. ^+^Others included village doctor, security guard, banker and worker. ^‡^Others included farmer

Table 2 shows the comparison of MMP-2 and MMP-9 concentrations between the study populations in arsenic-endemic and non-endemic areas. The average (Geometric mean ± GSD) value of MMP-2 and MMP-9 concentrations in the population group in arsenic-endemic areas were 225.20 ± 1.40 ng/mL and 54.43 ± 1.73 ng/mL, respectively, while these were 191.90 ± 1.50 ng/mL and 41.14 ± 1.56 ng/mL respectively, in the population group in non-endemic area. Both MMPs were found to be significantly (*p* < 0.001 for both MMPs) higher in arsenic-endemic population than in non-endemic population.

**Table 2:** Comparisons of Serum MMP-2 and MMP-9 concentrations between the study populations in arsenic-endemic and non-endemic areas

Parameters	Non-endemic	Arsenic-endemic	*p*-value
Serum MMP-2 (ng/mL)^a^	191.90 (1.50)	225.20 (1.40)	<0.001
	(*n* = 108)	(*n* = 261)	
Serum MMP-9 (ng/mL)^a^	41.14 (1.56)	54.43 (1.73)	<0.001
	(*n* = 108)	(*n* = 265)	

^a^Geometric Mean (GSD). Log-transformed dependent variables were used. *p*-values were from the Independent sample t-test

Table 3 shows the correlations of arsenic exposure metrics (water, hair and nail arsenic concentrations) with serum MMP-2 and MMP-9 concentrations. Serum concentrations of MMP-2 and MMP-9 showed significant positive correlations with arsenic in water (*r*_*s*_ = 0.208, *p* < 0.001 for MMP-2; *r*_*s*_ = 0.163, *p* < 0.01 for MMP-9), hair (*r*_*s*_ = 0.163, *p* < 0.01 for MMP-2; *r*_*s*_ = 0.173, *p* < 0.01 for MMP-9) and nails (*r*_*s*_ = 0.160, *p* < 0.01 for MMP-2; *r*_*s*_ = 0.182, *p* < 0.001 for MMP-9). Dose–response relationships were tested into three arsenic exposure groups of the study subjects: low (non-endemic), medium and high (Table 4). Intriguingly, both MMP-2 and MMP-9 concentrations were found to be increased in the higher exposure gradients of water, hair and nail arsenic concentrations. MMP-2 and MMP-9 concentrations were 1.02 and 1.03 times greater, respectively, for every 1-unit increase in log-transformed water arsenic (μg/L), and were 1.03 and 1.06 times greater, respectively, for 1-unit increase in log-transformed hair arsenic concentration (μg/g) after adjusting for age, sex, BMI, smoking and hypertension. For 1-unit increase in log-transformed nail arsenic concentration (μg/g), MMP-2 and MMP-9 concentrations were 1.05 and 1.07 times greater, respectively. Further study populations were divided into three (≤10 μg/L, 10.1-50 μg/L and > 50 μg/L) groups based on the regulatory upper limit of water arsenic concentrations set by WHO (10 μg/L) and Bangladesh Government (50 μg/L), and then serum MMP-2 and MMP-9 concentrations were compared in these three groups (Table 5). Both MMPs were found to be gradually increased in the higher gradients compared to the lower gradients of water arsenic concentrations, however, significant (*p* < 0.01 and *p* < 0.001 for MMP-2 and MMP-9, respectively) differences were only observed in > 50 μg/L versus ≤ 10 μg/L groups.

**Table 3:** Correlations of arsenic exposure with serum MMP-2 and MMP-9 concentrations

Exposure metrics	MMP-2 (*n* = 369)		MMP-9 (*n* = 373)	
	Correlation coefficient (*r*_*s*_)	*p*-value	Correlation coefficient (*r*_*s*_)	*p*-value
Water arsenic	0.208	<0.001	0.163	<0.01
Hair arsenic	0.163	<0.01	0.173	<0.01
Nail arsenic	0.160	<0.01	0.182	<0.001

Log-transformed values were used*. p-*values were from Spearman correlation coefficient test

**Table 4:** Associations of arsenic exposure with serum concentrations of MMP-2 and MMP-9

Water Arsenic (μg/L)				
	Low ( 0.03–13.17)	Medium (0.11–133.8)	High (134.12–546)	Per log-transformed water As (μg/L)
	(*n* = 108)	(*n* = 133)	(*n* = 132)	
	Exp (B) (95 % CI)	Exp (B) (95 % CI)	Exp (B) (95 % CI)	Exp (B) (95 % CI)
GM (GSD)^a^	0.56 (7.78)	21.33 (5.47)	274.51 (1.51)	-
MMP-2				
Model 1	1.00 Reference group	1.14 (1.04–1.25)	1.20 (1.10–1.32)	1.02 (1.01–1.04)
Model 2	1.00 Reference group	1.12 (1.03–1.23)	1.20 (1.09–1.31)	1.02 (1.01–1.04)
GM (SE)^d^	191.90 (1.03)	219.64 (1.03)^f**^	230.67 (1.03)^f***^	
Adj GM (SE)^d^	201.74 (1.05)	227.01 (1.04)^f*^	241.53 (1.04)^f**^	
MMP-9				
Model 1	1.00 Reference group	1.32 (1.15–1.50)	1.33 (1.17–1.52)	1.03 (1.01–1.05)
Model 2	1.00 Reference group	1.28 (1.12–1.46)	1.26 (1.13–1.48)	1.03 (1.01–1.05)
GM (SE)^e^	41.14 (1.05)	54.22 (1.05)^f***^	54.65 (1.05)^f**^	
Adj GM (SE)^e^	45.60 (1.07)	58.32 (1.06)^f**^	59.15 (1.06)^f**^	
Hair Arsenic (μg/g)				
	Low (0.02–1.62)	Medium (0.01–2.84)	High (2.86–37.24)	Per log-transformed hair As (μg/g)
	(*n* = 108)	(*n* = 133)	(*n* = 132)	
	Exp (B) (95 % CI)	Exp (B) (95 % CI)	Exp (B) (95 % CI)	Exp (B) (95 % CI)
GM (GSD)^b^	0.23 (2.36)	1.28 (2.28)	6.4 (1.91)	-
MMP-2				
Model 1	1.00 Reference group	1.14 (1.04–1.25)	1.21 (1.10–1.32)	1.03 (1.009–1.06)
Model 2	1.00 Reference group	1.13 (1.03–1.24)	1.20 (1.09–1.31)	1.03 (1.006–1.055)
GM (SE)^d^	191.90 (1.03)	219.2 (1.03)^f*^	231.6 (1.03)^f***^	
Adj GM (SE)^d^	201.34 (1.05)	227.24 (1.04)^f*^	241.05 (1.04)^f**^	
MMP-9				
Model 1	1.00 Reference group	1.29 (1.13–1.47)	1.36 (1.19–1.55)	1.07 (1.03–1.11)
Model 2	1.00 Reference group	1.26 (1.10–1.44)	1.32 (1.15–1.51)	1.06 (1.03–1.10)
GM (SE)^e^	41.14 (1.05)	53.04 (1.05) ^f**^	55.87 (1.05) ^f***^	
Adj GM (SE)^e^	45.60 (1.07)	57.40 (1.06)^f**^	60.16 (1.06) ^f**^	
Nail Arsenic (μg/g)				
	Low (0.03–8.13)	Medium (0.22–6.79)	High (6.91–47.83)	Per log-transformed nail As (μg/g)
	(*n* = 108)	(*n* = 133)	(*n* = 132)	
	Exp (B) (95 % CI)	Exp (B) (95 % CI)	Exp (B) (95 % CI)	Exp (B) (95 % CI)
GM (GSD)^c^	0.80 (2.64)	3.21 (1.93)	13.65 (1.54)	-
MMP-2				
Model 1	1.00 Reference group	1.14 (1.04–1.25)	1.20 (1.10–1.32)	1.06 (1.03–1.09)
Model 2	1.00 Reference group	1.13 (1.03–1.23)	1.20 (1.09–1.32)	1.05 (1.03–1.08)
GM (SE)^d^	191.90 (1.03)	219.2 (1.03) ^f*^	231.60 (1.03) ^f***^	
Adj GM (SE)^d^	200.34 (1.05)	225.65 (1.04)^f*^	240.33 (1.04) ^f**^	
MMP-9				
Model 1	1.00 Reference group	1.30 (1.14–1.48)	1.35 (1.18–1.54)	1.08 (1.04– 1.12)
Model 2	1.00 Reference group	1.28 (1.12–1.47)	1.29 (1.13–1.48)	1.07 (1.02–1.11)
GM (SE)^e^	41.14 (1.05)	53.04 (1.05)^f**^	55.87 (1.05)^f**^	
Adj GM (SE)^e^	45.56 (1.07)	58.38 (1.06)^f**^	58.97 (1.06) ^f**^	

*n* = number of study subjects. Study subjects in the non-endemic area were used as low exposure group or reference group. Model 2 adjusted for age, sex, BMI, smoking and hypertension. Log transformed dependent and independent variables were used. ^a^Category specific geometric mean (GSD) values of water arsenic. ^b^Category specific geometric mean (GSD) values of hair arsenic. ^c^Category specific geometric mean (GSD) values of nail arsenic. ^d^Category specific geometric mean (SE) values of MMP-2. ^e^Category specific geometric mean (SE) values of MMP-9. ^f^Significantly different from low group. ***, *p* < 0.001; **, *p* < 0.01; *, *p* < 0.05

**Table 5:** MMP-2 and MMP-9 concentrations in three groups based on the regulatory limit of water arsenic

Parameters	≤10 μg/L	10.1–50 μg/L	>50 μg/L	*p*-value (F-test)
Serum MMP-2 (ng/mL)^a^	197.75 (1.48)	220.08 (1.37)	228.38 (1.40)^**^	<0.01
	(*n* = 144)	(*n* = 44)	(*n* = 181)	
Serum MMP-9 (ng/mL)^a^	43.60 (1.59)	53.57 (1.75)	55.20 (1.75)^***^	<0.001
	(*n* = 145)	(*n* = 44)	(*n* = 184)	

*n* = number of study subjects. ^a^Geometric Mean (GSD). ^**^Significantly different from ≤ 10 μg/L group at *p* < 0.01. ^***^Significantly different from ≤ 10 μg/L group at *p* < 0.001. *p*-values were from one way ANOVA test

**Fig. 1:**
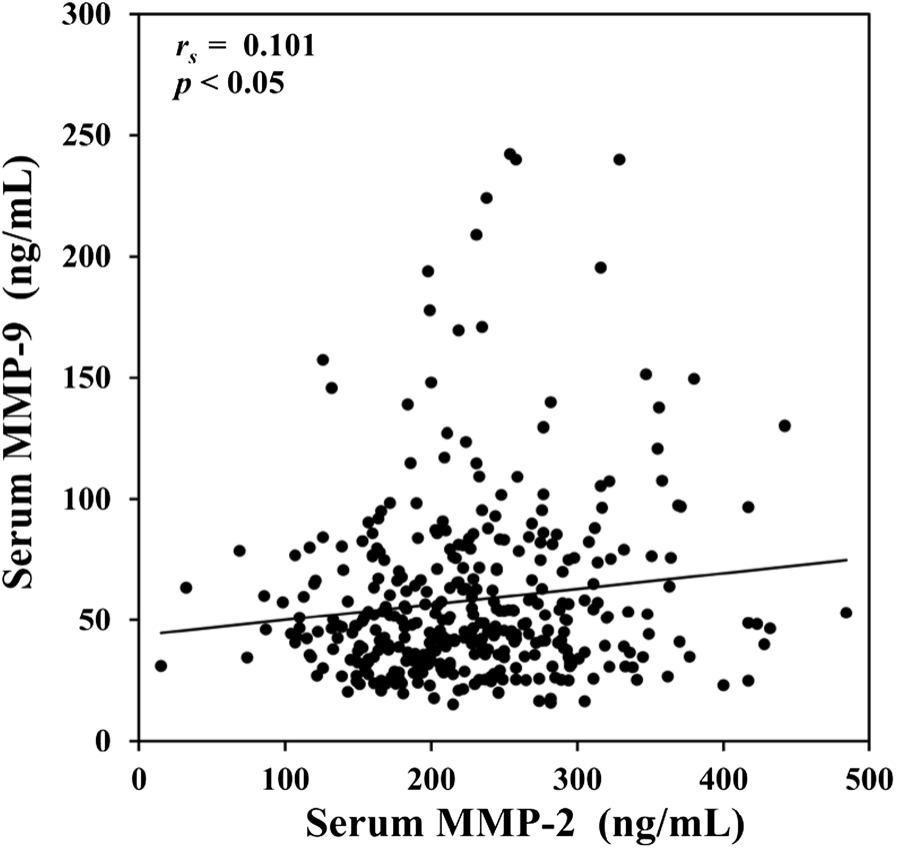
Correlation between serum MMP-2 and MMP-9 concentrations. *p-*value was from Spearman correlation coefficient test

Since serum MMP-2 and MMP-9 concentrations showed positive associations and dose–response relationships with arsenic exposure, we next investigated the interrelationship of these two MMPs. Intriguingly, we found that MMP-2 and MMP-9 concentrations were significantly (*p* < 0.05) correlated with each other (Fig. 1).

We have previously investigated the associations of arsenic exposure with several circulating markers of CVDs such as high density lipoprotein cholesterol (HDL-C), intercellular adhesion molecule-1 (ICAM-1) and vascular cell adhesion molecule-1 (VCAM-1) on the same population group selected for this study [3]. Positive associations of MMP-2 and MMP-9 concentrations with arsenic exposure led us to analyze the relationship of both MMPs with cardiovascular markers among the study subjects who were common for the both previous and present studies (Table 6). A good number of study subjects in this study (*n* = 303 for HDL-C; *n* = 313 for ICAM-1; *n* = 314 for VCAM-1) were overlapped with our previous study [3]. MMP-9 but not MMP-2 showed the significant (*p* < 0.01) inverse relationship with plasma concentrations of HDL-C of the overlapping study subjects of the two studies. Furthermore, significant positive associations were observed between MMP-2 and VCAM-1 (*p* < 0.05), between MMP-9 and VCAM-1 (*p* < 0.05), and between MMP-9 and ICAM-1 (*p* < 0.01) concentrations.

**Table 6:** Correlations of MMP-2 and MMP-9 with HDL-C, ICAM-1 and VCAM-1

		HDL-C	ICAM-1	VCAM-1
MMP-2	*r*_*s*_	−0.071	0.103	0.133
	*p*-value	0.211	0.068	0.019
	n	303	313	314
MMP-9	*r*_*s*_	−0.178	0.157	0.130
	*p*-value	0.002	0.005	0.021
	n	303	313	314

*n* = number of study subjects overlapped with previous study [3]. *p-*values were from Spearman correlation coefficient test

## Discussion

In this cross sectional study, serum MMP-2 and MMP-9 concentrations were found to be significantly higher in arsenic-endemic population than those in non-endemic population in rural Bangladesh (Table 2). Arsenic exposure measured by drinking water (external exposure metric), hair and nail (internal exposure metrics) showed positive interactions and dose response relationship with serum MMP-2 and MMP-9 concentrations (Tables 3, 4 and 5). Both MMPs were also significantly correlated with each other (Fig. 1). Finally, elevated concentrations of serum MMP-9, and to a lesser extent MMP-2, were found to be linked to the several circulating biomarkers (HDL-C, ICAM-1 and VCAM-1) associated with the risk of CVDs (Table 6).

MMPs act as inflammatory mediators [28]. The major cellular sources of MMPs are macrophages and neutrophils. MMPs degrade ECM to facilitate migration and recruitment of cells including inflammatory cells, and cleave cell surface receptors and non-ECM molecules to mediate adhesion, proliferation and apoptosis of cells in vessel wall which are involved in the inflammatory process [29, 30]. Growing evidence suggests that MMP-2 and MMP-9 are deeply implicated in CVDs and cancers through vascular remodeling and the formation of new blood vessels (angiogenesis and arteriogenesis) [31–34]. In matured and quiescent vessels, small amount of MMP-2 and MMP-9 are expressed. But when tissue undergoes vascular remodeling and abnormal angiogenesis in pathological conditions especially in CVDs and cancer, MMPs are markedly expressed, secreted and activated. Atherosclerosis is the fundamental event for CVDs. Formation of atherosclerotic plaque, and its progression and rapture are largely mediated through inflammatory process [35]. MMPs take part in the early stage of atherosclerotic process by enhancing migration and proliferation of smooth muscle cells as well as other inflammatory cells, whereas in advanced stage of atherosclerosis, inflammation derived proteolytic activity of MMPs may weaken and rapture the plaque [14]. Wågsäter et al. [36] investigated several MMPs, other proteases and TIMP-1, and found that only MMP-2 and MMP-9 were involved in atherogenesis in Ldlr^−/−^Apob100/100 mouse model which had a plasma lipoprotein profile similar to that of humans with atherosclerosis. In this mouse model, atherosclerotic lesion development was found to be initiated by infiltrated macrophage that mainly produced MMP-2 and MMP-9.

There have been discrepancies in the association of arsenic exposure with MMP-2 and MMP-9 concentrations from one study to another. The results of this study are in good agreement with the results reported by Burgess et al. [16]. They found a positive correlation of MMP-9 concentrations with water arsenic concentrations of the total population that include Mexicans, US Hispanic and US Non-Hispanic individuals. A cross sectional study in two communities in Arizona reported an effect of low concentration of arsenic exposure on MMP-2, MMP-9 and their inhibitor TIMP-1 [17]. This study did not find any significant differences in sputum MMP-2, MMP-9 and TIMP-1 concentrations in the relatively high exposed population as compared to the low exposed population. However, this study showed the negative associations of total urinary arsenic with MMP-2 and TIMP-1, and positive associations with the ratios of sputum MMP-2/TIMP-1 and MMP-9/TIMP-1. Findings of our study were different as compared with the study conducted on the population in arsenic-endemic area in Bangladesh by Wu et al. [18]. Wu’s study did not find any significant positive association between arsenic exposure and plasma MMP-9 concentrations. There were several important differences in the characteristics of the study populations between our study and Wu’s study. Arsenic concentrations in the drinking water of our study subjects were much higher than those of the study subjects selected for Wu’s study. In our study, 14.48 % of individuals were exposed to tube well water arsenic at concentrations greater than 300 μg/L (data not shown), whereas 98 % of the study subjects of Wu’s study were exposed to water arsenic at concentrations less than 300 μg/L. Further 49.33 % of our study subjects had arsenic-induced typical skin lesions (melanosis, leukomelanosis and keratosis), whereas only 3.4 % of individuals of Wu’s study had skin symptoms. Therefore, exposure to high concentrations of arsenic and prevailing typical skin symptoms in a good number of study subjects are the major characteristic features of our study population that might make differences in the results of our study from Wu’s study.

Our study showed not only associations but also dose–response relationships of arsenic exposure with serum MMP-2 and MMP-9 concentrations. MMP-2 concentrations were 1.02, 1.03 and 1.05 times, and MMP-9 concentrations were 1.03, 1.06 and 1.07 times greater for 1 unit increase in log-transformed water, hair and nail arsenic concentrations, respectively (Table 4). In addition, both MMPs were found to be increased dose-dependently when the study subjects were split into three groups based on the regulatory upper limit of water arsenic concentration set by WHO and Bangladesh Government (Table 5). In this study, hair and nail arsenic concentrations corresponded well to the drinking water arsenic concentrations of the study subjects (data not shown) as we reported previously [23, 24]. These strong interrelationships among exposure metrics could reduce the bias in the observed associations of arsenic exposure with MMPs. Wide variation of exposure concentrations, good number of study population, consistent associations of MMP-2 and MMP-9 concentrations across the three kinds of exposure metrics (drinking water, hair and nail arsenic), and the significant interrelationship between the two MMPs are the main strengths of this study. Previous studies used drinking water and urinary arsenic concentrations as exposure metrics to demonstrate the associations of arsenic exposure with MMPs [17, 18]. Urinary arsenic does not reflect the historical or chronic exposure. Arsenic concentrations in biologic tissues other than nails and hair, such as blood or urine reflect recent exposure. Arsenic is removed from the blood within a few hours and excreted through the kidneys and urine within a few days [37, 38]. Nail arsenic has been suggested to be an indicator of historical or chronic exposure [39]. Hair arsenic concentrations also represent historical exposure but compared to nail, it represents relatively immediate exposure. One centimeter of hair from the stem reflects approximately one month of exposure. Therefore, assessment of the effects of chronic arsenic exposure measured by hair and nail arsenic on serum MMP-2 and MMP-9 concentrations was an additional strength of this study.

We selected the study subjects for this study who had also attended the previous studies in which association of arsenic exposure with circulating markers of CVDs were investigated [3]. Therefore, positive associations of arsenic exposure with serum MMP-2 and MMP-9 concentrations on the same population group argued their implications in the arsenic-induced cardiovascular pathogenesis. This argument was further supported by the observed associations of MMP-2 and MMP-9 concentrations with some biomarkers related to CVDs (Table 6). MMP-9 showed significant (*p* < 0.01) negative association with HDL-C. Decreased serum HDL-C concentration is a traditional biomarker of the risk of atherosclerosis leading to CVDs. Significant negative relationship of serum MMP-9 with HDL-C indicated the implication of serum MMP-9 concentrations in the development of atherosclerosis in arsenic-affected people. Accumulating evidence suggests that systemic inflammation and endothelial dysfunction are two hallmark events of the development of CVDs [40, 41]. Previously, on the same populations, we found that arsenic exposure increased endothelial dysfunction that was determined by plasma big-endothelin-1 (Big ET-1) concentrations [22]. Activated endothelial cells express adhesion molecules that mediate the attachment of circulating leukocytes to the endothelium and their transmigration into the arterial wall in the early step of atherosclerosis. Circulating adhesion molecules including ICAM-1 and VCAM-1 have been reported to be markers of future risk of CVDs [42, 43]. Previously, we and others have also showed that arsenic exposure increased the circulating ICAM-1 and VCAM-1 [3, 18, 44]. More than 300 individuals of this study were overlapped with our previous study in terms of ICAM-1 and VCAM-1. Intriguingly, we found significant positive associations between MMP-2 and VCAM-1, between MMP-9 and ICAM-1, and between MMP-9 and VCAM-1. Therefore, elevated concentrations of MMP-2 and MMP-9 observed in this study strongly argued the involvement of these proteases in the pathophysiology of arsenic-induced CVDs.

Although this study represents extensive epidemiological research efforts to determine the effects of arsenic exposure on serum MMP-2 and MMP-9 concentrations, there were some potential limitations warranting further discussion. One of the limitations of this study was to assess only the serum MMP-2 and MMP-9 concentrations but not their activities. MMP expression and activity do not always correlate each other. Enzymatic activities of MMPs are directly relevant to the biological functions. Activities of serum TIMPs have also putative effects on MMP activity. Previously Josyula et al. [17] have showed an inverse relation between urinary inorganic arsenic and sputum TIMP-1. Several *in vitro* studies have also demonstrated that arsenic exposure increased the activities of MMP-2 and MMP-9 [45–47]. Therefore, it is assumed that increased concentrations of MMP-2 and MMP-9 with the increasing concentration of arsenic observed in this study may correspond to the activities of both MMPs. To assess the effects of other variables in the observed associations of arsenic exposure with serum MMPs, adjustments were made for covariates (age, sex, BMI, smoking habit and hypertension), and the results explicitly demonstrated that arsenic exposure was the main contributor to the increasing concentrations of MMP-2 and MMP-9 in serum (Table 4). However, there might be some other variables or co-exposure of other metals that could influence the observed associations. If co-exposure of other metals influenced the observed associations, they would follow the same concentration gradients as arsenic did. It is unlikely; however, probabilities could not be excluded completely. Future study is needed in this regard. The study design used here was a cross-sectional. A cohort based study is needed in future to establish the cause-effect relationship between arsenic exposure and serum MMPs. Most of our study subjects in both arsenic endemic and non-endemic areas were lean with regard to BMI. Furthermore, about half of the study subjects had typical symptoms of arsenicosis. These characteristics of the study population may limit the generalizability of the findings of this study.

## Conclusions

This study demonstrated that serum MMP-2 and MMP-9 concentrations in the study subjects were significantly higher in arsenic-endemic areas than in non-endemic area. Increased serum MMP-2 and MMP-9 concentrations showed significant associations with the increasing concentrations of drinking water, hair and nail arsenic of the study subjects. Arsenic exposure exhibited dose–response relationships with serum MMP-2 and MMP-9 concentrations. Furthermore, both MMPs were positively correlated with each other. Finally, serum MMP-2 and MMP-9 concentrations showed associations with the several circulating markers of CVDs. Thus, the results of this study suggest that arsenic exposure-related elevation of serum MMP-2 and MMP-9 concentrations may be implicated in arsenic-induced CVDs.

## Abbreviations

CVDs: cardiovascular diseases
ECM: extracellular matrix
MMPs: matrix metalloproteinases
